# The Interplay of Compassion, Subjective Happiness and Proactive Strategies on Kindergarten Teachers’ Work Engagement and Perceived Working Environment Fit

**DOI:** 10.3390/ijerph17134869

**Published:** 2020-07-06

**Authors:** Simona De Stasio, Paula Benevene, Alessandro Pepe, Ilaria Buonomo, Benedetta Ragni, Carmen Berenguer

**Affiliations:** 1 Department of Human Studies, LUMSA University, 00193 Rome, Italy; benevene@lumsa.it (P.B.); i.buonomo1@lumsa.it (I.B.); b.ragni@lumsa.it (B.R.); 2 Department of Human Sciences “Riccardo Massa”, Bicocca University, 20126 Milan, Italy; alessandro.pepe1@unimib.it; 3 Department of Developmental and Educational Psychology, University of Valencia, 46010 Valencia, Spain; carmen.berenguer@uv.es

**Keywords:** kindergarten teachers, compassion, subjective happiness, working environment fit, proactive strategies, work engagement

## Abstract

Background: The current cross-sectional study examines a model that was designed to advance understanding of the interplay between compassion towards teachers expressed by teaching colleagues, subjective happiness, proactive strategies and kindergarten teachers’ levels of work engagement, and perceived working environment fit. Methods: The research was conducted with a sample of 319 full-time in-service kindergarten teachers at Italian public preschools—a context in which a few previous studies have been carried out. Self-report questionnaires were administered: The Subjective Happiness Scale, the Santa Clara Brief Compassion Scale, the Utrecht Work Engagement Scale, the Proactive Strategy Scale, and the Teacher-working environment fit scale. Data were analyzed by using the structural equation modelling (SEM) approach. Results: Results show that compassion and subjective happiness have a direct positive total effect on work engagement, whereas the effects of compassion and subjective happiness on experienced working environment fit suggest that the association among constructs is mediated by the role of proactive strategies. Conclusions: Based on these findings, we strongly advocate that educational policy makers and head teachers’ pay close attention to the areas of personal and collective resources and work-related well-being, with a view to effectively address the promotion of early childhood teachers’ work engagement and working environment fit.

## 1. Introduction

Kindergarten teachers care for children between 3 and 6 years old. This requires advanced emotional competence in order to deal with everyday emotional situations at school, remain relationally connected with children and parents, and cooperate with colleagues [1,2,3]. Indeed, kindergarten teachers, unlikely their colleagues of the primary and secondary schools, are required to perform a range of caring activities in addition to their educational tasks [1,4,5] and to work in teams, to better address the complexity of kindergarten tasks and demands by means of a constant contact between teaching colleagues, head teachers, and members of other workgroups [6]. These practices and strategies are officially required to Italian kindergarten teachers: according to OECD reports [7], indeed, guidelines for kindergarten teachers focus on three dimensions: (1) teacher’s contribution to children’s emotional and intellectual growth, by means of social relationships; (2) the role of kindergarten as an informal transition from family context to primary school; and (3) the importance of home–school relations.

Given the highly relational nature of the early childhood work, the current study was designed to examine the potential interdependence between teacher subjective happiness, compassion from teaching colleagues, and key work-related outcomes (such as work engagement and work environment fit) with a view to inform training programs for enhancing resilience and preventing professional dropout in kindergarten teachers.

Work engagement, the first of these outcome variables, has been defined as “a state of fulfilment that is characterized by vigor, dedication, and absorption” [8]. Previous studies have established that higher levels of work engagement predict stronger job performance and reduced intention to quit teaching, as well as wielding a “positive contagion” effect on teacher–student relationships and pupils’ academic achievement [9,10,11].

The second outcome, namely, experienced work environment fit, investigated in relation to teachers, comprises two core dimensions: professional recognition from colleagues and a positive, constructive, and fair work climate [12]. Previous studies specifically informed on the first dimension, showing that receiving constructive feedback and professional recognition at school [13,14,15] foster job satisfaction and work engagement which, in turn, improve job performance [16,17].

Considered the implications of both work engagement and work environment fit for teacher performance, as well as the relational nature of early childhood teachers’ work, it is of interest to investigate how dimensions such as compassion and subjective happiness may contribute to work engagement and experienced work environment fit.

Existing research has pointed up a positive association between benefiting from compassion in the workplace and enhanced work engagement and productivity [18]. Furthermore, when people receive compassion from others at work, they perceive their colleagues as more humane and their organization as more caring [19]. Regarding the educational context, the few existing studies show that teachers receiving compassion not only have better relationships with their colleagues but show higher work engagement and fit with the organization [20,21,22].

Previous research has shown a positive relationship among subjective happiness and positive work-related outcomes. Generally speaking, subjective happiness is usually associated with job satisfaction and work engagement [23]. Happy people, indeed, perform better and help their colleagues more often than do their coworkers who are less happy [24]. At the same time, studies on teachers showed that high subjective happiness is associated to positive outcomes at work, such as positive climate long-term well-being at work, self-efficacy beliefs, and job satisfaction [25].

Despite previous works inform us about the positive impact of subjective happiness on work outcomes, there is still a lack of research on how receiving compassion from others and experiencing subjective happiness may influence kindergarten teachers’ work engagement.

Drawing on Fredrickson’s [26] broaden-and-build theory of positive emotion, we hypothesized that frequent positive affect (PA), underpinned by experiencing subjective happiness and benefiting from compassion at work, would influence work outcomes in teachers. According to the broaden-and-build theory, experiencing positive emotions at work broadens individuals’ mindsets and helps them to build on their personal resources [27,28], such as their sensitivity to opportunity, openness, and positive attitudes towards their workplace [29,30,31,32]. Thus, frequent positive affect as a result of receiving compassion from peers may help teachers to form pleasant and positive emotional associations with their school, gradually reinforcing their emotional vigor, organizational commitment, and experienced working environment fit [20,26,33].

### 1.1. Compassion and Subjective Happiness as Antecedents of Work Engagement

Compassion and subjective happiness are key personal resources that studies suggest may be associated with engagement in the workplace [34,35].

As noted above, work engagement encompasses three dimensions: vigor, dedication, and absorption [8]. More specifically, vigor denotes energy and the willingness to put in extra effort and be persistent in one’s work; dedication implies being deeply involved in one’s job and experiencing a sense of meaningfulness, enthusiasm, and inspiration; absorption means being fully focused on and happily engrossed in one’s work, such that time passes quickly [36].

Again, teachers who display a higher level of engagement of their colleagues, tend to perform better, are less likely to intend leaving teaching, and enjoy more positive relationships with their students, who, in turn, are higher academic achievers [9,11,37].

Subjective happiness is related to work engagement: individuals who are happier with their lives are also happier with their work. They display lower turnover rates, a greater capacity to invest energy in their jobs, and more frequent positive emotion, all of which, in turn, generate engagement [24,37,38].

Surprisingly, there is still a lack of research on the association between happiness and work engagement in schools [39,40,41].

More specifically, subjective happiness at work has typically been studied as an outcome of work engagement. Although other research has focused on personal resources such as resilience and optimism as antecedents of work engagement, to the best of our knowledge, there are no studies on the effect of happiness on work engagement in teachers [17,42]. Furthermore, no existing research has examined this relationship in kindergarten teachers [43,44].

### 1.2. Compassion and Subjective Happiness as Antecedents of Experienced Work Environment Fit

Compassion and subjective happiness are also associated with experienced work environment fit [45,46,47,48], which is defined as self-perceived contextual congruence or dissonance between one’s personal characteristics and those of one’s organizational setting [49].

This factor can have major implications for how people relate to their working environment. For example, contextual dissonance can lead to the feeling that there is something wrong with one’s own organization which, in turn, may generate dissatisfaction and withdrawal [50]. In the case of teachers (see the earlier cited study by Pyhältö et al. [12]), work environment fit is a function of professional recognition from teaching peers and a positive, constructive, and fair working atmosphere.

An association between compassion and work environment fit has been identified among nurses but remains virtually unexplored in relation to other helping professionals [51]. Yet compassion is a dispositional trait of great value in helpers. Compassion among practitioners generates healing energy which flows back to those who offer it, setting off a virtuous cycle and reinforcing helpers’ sense of congruence with their work setting [52].

Like compassion, subjective happiness is implicated in organizational fit. Park and colleagues reported that employees reporting higher levels of work environment fit also reported greater happiness [53]. Despite this, to the best of our knowledge, the relationship between these two variables remains to be fully explored, especially among teachers.

### 1.3. The Role of Proactive Strategies

The effects of dispositional resources such as subjective happiness and compassion on how teachers experience their work may be enhanced by using proactive strategies. Indeed, individuals’ quality of interaction with their work environment can either help or hinder them in addressing the challenges of their jobs [12,54,55].

The teachers can adopt strategies that help them cope with demanding situations and buffer the effects of work stress [12,54]. The strategies learned and used by teachers differ in terms of reactivity and proactivity. When facing potentially stressful situations, teachers can use reactive coping strategies, i.e., they can react to the stressor by regulating their feelings or altering the situation so that the stress is diminished [56]. Proactive strategies (both self-regulated and coregulated) involve preventing or acting pre-emptively to mitigate potentially stressful events, thus neutralizing the stressor before it becomes harmful [54,55,57,58,59].

More specifically, self-regulated strategies are self-generated behavioral, cognitive, and emotional responses that individuals deploy to cope more effectively with stressors; they can include better planning, seeking out new information, learning new skills, and reducing work tasks that feel burdensome [58].

On the basis of the earlier cited broaden-and-build theory of positive emotions [26], it is possible to argue that experiencing frequent positive affects, related in the school context, e.g., sharing and discussing work-related concerns with colleagues, can enable individuals to broaden their cognitive and behavioral repertoires, and thereby contribute to increasing of their personal resources such as self-efficacy, resilience, and optimism The relevance of addressing such strategies lies in the fact that the learning and the use of them depend on the social interaction of the schools where the teachers work, therefore, they can be intentionally promoted and sustained [54].

Proactive coregulated strategies involve contributing to and drawing on the social resources available within the workplace; examples include asking for and receiving, emotional and informational support from colleagues [55,60] as well as sharing and discussing work-related concerns [61].

It is of value to investigate coregulated strategies because they are learnt and deployed in the course of social interaction among teaching staff and can therefore be intentionally fostered and supported [54].

There are evidences that the use of proactive strategies is positively associated with teachers’ work environment fit [54,55,62], as well as with their work engagement.

Thus, adopting proactive strategies may not only reduce teachers’ risk of burnout but also contribute to develop a positive working environment and increase work engagement. However, to the best of our knowledge, the role of the different strategies adopted by kindergarten teachers to generate work engagement and person–organization fit, have yet to be studied in depth.

### 1.4. The Current Study

The present quantitative multitrait cross-sectional study aims to gain a better understanding of the interplay between subjective happiness, compassion, levels of work engagement, perceived working environment fit, and proactive strategies (self-regulated and coregulation) in a sample of kindergarten teachers.

In keeping with the current literature, we would expect to find: that subjective happiness and compassion of kindergarten teachers would be related with work engagement and perceived working environment fit (H1).

In a similar fashion, we theorized that subjective happiness would be positively related to self-regulated and coregulation strategies (H2).

The proactive strategies would be in turn associated to both work engagement and experienced working environment fit (H3)

Finally, and most germane for the present paper, the representation of the network of association (i.e., total, direct, and indirect effects) in an integrated structural model supported the theoretical viewpoints [63] considering subjective happiness, work engagement, and environmental working condition (and fit) as dynamically shaping the social endeavor in which kindergarten teachers are involved during their daily profession. It must be stated here that, in adopting the term “expected effects,” we referred to the assessment of direct natural effects [63], which allows for natural variation between subjects in the level of the target variables rather than interpreting a direct effect from the lens of causality framework. With regard to indirect effects between considered variables, no specific hypotheses were formulated.

## 2. Materials and Methods

Our sample was composed of 319 full-time in-service teachers (92.7% female) in public kindergartens of Rome, Italy. Ages ranged from 24 to 66 years (M = 42.26 years, SD = 16.87). In terms of marital status, 55.8% were married, 15.8% were single, 23.3% were separated/divorced, and 1.6% were widowed. In total, 66.6% of participants had children. Length of teaching experience ranged from 1 to 42 years (M = 17.23 years, SD = 14.23).

The study population was a convenience sample and may not be taken as representative of the entire population of Italian teachers given that all participants were based in Central Italy. The authors organized plenary assemblies in schools to inform the teachers about the aims of the research and the procedures for completion of the questionnaires. Participants received written information on Italian privacy regulations, signed informed consent, and subsequently took part in the study. The research was conducted following the APA’s ethical principles and code of conduct [64]. The study was approved by the Ethics Committee for Scientific Research of LUMSA University of Rome, Italy. The original versions of questionnaires were initially translated from English into Italian and then back translated into English to check the alignment with the original versions.

### 2.1. Measures

#### 2.1.1. Subjective Happiness Scale

The Subjective Happiness Scale [65] is a 4-item scale aimed at assessing subjective happiness, using a 7-point Likert scale. The first two items ask people to rate how they are generally happy about their life (1 = *not a very happy person* and 7 = *a very happy person*) and how happy they are in comparison with their peers (1 = *less happy* and 7 = *more happy*); the last two items ask respondents to what extent the characterization of an happy and of an unhappy person describe themselves (1 = *not at all* and 7 = *a great deal*). Higher scores on this measure indicate greater subjective happiness. Prior studies have reported Cronbach’s alpha coefficients for the SHS from 0.79 to 0.94 [65]. In the current study, reliability for SHS was 0.82.

#### 2.1.2. Santa Clara Brief Compassion Scale

The Santa Clara Brief Compassion Scale [66] is a 5-item scale; it assesses compassion and its link to prosocial behaviors. The scale includes items related to two facets of compassion: “emotionally connecting with other people’s suffering” and “acting to help them.” It is a shortened version of Sprecher and Fehr’s [67] Compassionate Love Scale (the correlation between the two scales is *r* = 0.95) and it refers to nonintimate (i.e., strangers) rather than to close others. All items were rated on a 7-point Likert-type scale ranging from 1 (completely disagree) to 7 (completely agree) and higher scores are indicative of greater compassion. Some examples of the items used by the compassion scale were as it follows: (1) When I hear about someone (a stranger) going through a difficult time, I feel a great deal of compassion for him or her; (2) I tend to feel compassion for people, even though I do not know them; and (3) One of the activities that provide me with the most meaning to my life is helping others in the world when they need help. In the current study, reliability for the unidimensional version of the compassion scale was 0.89.

#### 2.1.3. Utrecht Work Engagement Scale

The Utrecht Work Engagement Scale (UWES-17, [68]) assesses work engagement. The scale is composed of 17 items, grouped into three subscales. Namely, vigor (six items), dedication (five items), and absorption (six items). All items are scored on a 7-point frequency rating scale ranging from 0 (never) to 6 (always). Examples of items included in the measure were as it follows: “At my work, I feel bursting with energy” (vigor); (2) “I am enthusiastic about my job” (dedication); (3) “I am immersed in my work” (absorption). In this study, the internal consistencies of different factors of the UWE-17 (Cronbach’s alpha; Cronbach, 1951) were vigor (α = 0.87), dedication (α = 0.88), and absorption (α = 0.85).

#### 2.1.4. Proactive Strategy Scale

The Proactive Strategy scale [69] consists of 7 items, measuring two factors of proactive strategies: (a) self-regulation (4 items) and (b) coregulation (3 items), meaning, respectively, the ability to identify and use resources for coping with stressors and the ability to seek and receive social support from colleagues. All items were rated on a 7-point Likert-type scale, ranging from 1 (completely disagree) to 7 (completely agree). Results of confirmatory factor analysis conducted on the pool of items, supported a two-dimensional solution for the model of measurement: χ^2^(13) = 38.7 and *p* < 0.05; NC = 2.97, RMSEA = 0.078, pclose = 0.044, NFI = 0.959, NNFI = 0.940, CFI = 0.972, and SRMR = 0.042. In this study, the internal consistencies of different factors of the teacher–working environment fit scale (Cronbach’s alpha; Cronbach, 1951) were self-regulation (α= 0.81) and coregulation (α= 0.76).

#### 2.1.5. Teacher-Working Environment Fit Scale

The teacher-working environment fit scale [12] consists of six items measuring two factors of experienced teacher-working environment fit: (a) received professional recognition (three items, e.g., My colleagues provide me with encouragement and support) and (b) constructive and enabling work climate (three items, e.g., My workplace has a fine atmosphere). Hence, the experienced fit was measured from both the individual and professional community perspectives. The professional recognition factor measured the individual teacher’s experienced appreciation as a member of a professional community (i.e., the person-centered approach to the perceived fit). The constructive and enabling work climate factor measured teachers’ shared capacity to contribute to the optimal fit within the professional community, as experienced by the respondent (i.e., the environment-centered approach to the perceived fit). All items were rated on a 7-point Likert scale ranging from 1 (completely disagree) to 7 (completely agree) [54]. Results of confirmatory factor analysis conducted on the pool of items supported a two-dimensional solution for the model of measurement: χ^2^(8) = 24.6 and *p* < 0.05; NC = 3.07, RMSEA= 0.079, pclose = 0.071, NFI = 0.970, NNFI = 0.946, CFI = 0.979, and SRMR = 0.048. In this study, the internal consistencies of different factors of the teacher–working environment fit scale (Cronbach’s alpha; Cronbach, 1951) were professional recognition (α = 0.85) and work climate (α = 0.77).

### 2.2. Strategy of Data Analysis

Data were analyzed by using the structural equation modelling (SEM) approach [70]. Such statistical technique can be seen as a generalization, integration, and extension of multivariate analysis of data based on analysis of covariance (e.g., MANOVA) and regression [71]. The SEM logic is based on the comparison between a hypothesized theoretical model (along with pattern of associations) and an empirical observed set of data. The evaluation of the degree of fit between the two models provided important information about its statistical and practical significance. Associations between variables can be read in term of magnitude and direction of observed phenomena. In the present study, the model was conceptualized by using subjective well-being (as measured by SHS), compassion (as measured by SCBCS), proactive strategies (as operationalized by self-regulated and coregulation strategies), working environment fit (as operationalized by climate and recognition), and work engagement (as measured by UWE).

Correlational analysis of data revealed that, in general, participants’ demographic variables (i.e., gender and age) were not statistically associated with the study measures. T-test suggested that male and female scores on variables of interest did not statistically differ. Only a small correlation between climate and age (*r* = 0.11, *p* < 0.01) was found. As a result, such variables were not included into the model following the principle of parsimony in data modelling [72]. In line with the tradition of studies adopting SEM approaches, the goodness of its indexes were evaluated, i.e., χ^2^ and normed-χ^2^ (NC) (a nonstatistically significant χ^2^ value and NC values of under 2.0 indicate good fit [73]); root mean square error of approximation (RMSEA), normed fit index (NFI), non-normed fit index (NNFI), and comparative fit index (CFI). Thresholds for model acceptance were: RMSEA < 0.07 [74], NFI > 0.95, NNFI > 0.95, and CFI > 0.95 [75]. In keeping with the current literature [76], we estimated 95% confidence limits using both Monte Carlo simulation and bootstrapping methods with a set of random samples (k = 500). We calculated given indirect effects for each of the k samples and the mean value for the selected pool of samples. Since the current study was based on self-reported quantitative measures, results can be subjected to Common Method Variance (CMV) bias. In order to test if CMV was a pervasive issue in our data, Harman’s Single-Factor Test d (i.e., a post hoc procedure that is conducted after data collection to check whether a single factor is accountable for variance in the data) was performed. The results of confirmatory factor analysis on the single latent factor structure (χ^2^(32) = 2074.5 and *p* < 0.05; NC = 2.46, RMSEA = 0.245, pclose < 0.001, NFI = 0.423, NNFI = 0.346, CFI = 0.956, and SRMR = 0.146) suggested a very poor fit meaning that the CMV bias was not a pervasive issue in the data and structural equation modelling can be used.

Finally, the following procedure of data exploration were applied: (a) univariate and multivariate outlier analysis (Mahalanobis distance was set to *p* < 0.001 [77]), (b) score distribution analysis (skewness and kurtosis cut-off points were set to [−2;+2] [78]) (c) missing value analyses (missing values were skipped listwise, Little, 1992). As a result, 7 (2.1%) outliers and 9 (2.8%) cases were excluded from the analysis. All variables reported scores resembling the normal distribution. The Maximum Likelihood method [79] was adopted to estimate the parameters for the SEM analysis.

## 3. Results

In Table 1, main descriptive statistics of all variables under study and Cronbach’s alpha coefficients for each instrument are summarized.

Bivariate correlations among the studied variables are presented in Table 2. Subjective happiness was significantly correlated with teachers’ self-regulated (*r* = 0.46) and coregulation strategies (*r* = 0.42), work engagement (*r* = 0.40), compassion (*r* = 0.33), and working environment fit (*r* = 0.13). Compassion was significantly correlated with work engagement (*r* = 0.38), teachers’ self-regulation (*r* = 0.26) and coregulation strategies (*r* = 0.27), and working environment fit (*r*= 0.22). Both teachers’ self-regulation (*r* = 0.48) and coregulation (*r* = 0.51) strategies were significantly correlated with work engagement. The teachers’ working environment fit was significantly correlated with work engagement (*r* = 0.37) and with teachers’ proactive strategies (self-regulation (*r* = 0.37) and coregulation (*r* = 0.47)).

Analysis of both absolute and relative fit indexes suggested that the conceptual model overlapped the structure of empirical data: χ^2^(91) = 224.5 and *p* < 0.05; NC = 2.46, RMSEA = 0.068, pclose = 0.005, NFI = 0.928, NNFI = 0.956, CFI = 0.956, and SRMR = 0.044.

**Figure 1:**
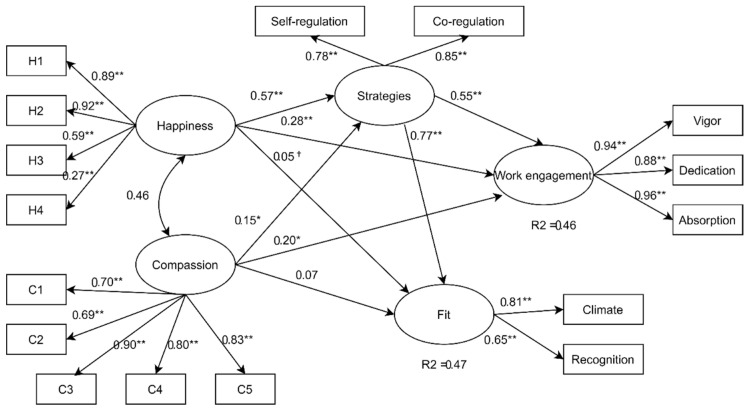
Results of the structural equation model. Association between happiness and compassion with teacher work engagement and fit. Happiness and compassion reported positive direct effects on the target variable with similar patterns but with different magnitudes. Note: Standardized direct effects were reported; * *p* < 0.05, ** *p* < 0.01, ^†^ not statistically significant.

The whole model fitted the data, and the results provided numerical support to the integrated view of early childhood teachers’ subjective well-being, compassion, proactive strategies, work engagement, and working environment fit (see the structural model in Figure 1).

With regard to the first hypothesis, the tested theoretical model revealed that subjective happiness showed a total positive effect on work engagement (β = 0.59, *p* < 0.01; C.I. 95% (1.00–1.98)) resulting from a direct effect (β = 0.28, *p* < 0.01; C.I. 95% (0.24–1.03)) and from a medium indirect effect via proactive strategies (β = 0.31, *p* < 0.01; C.I. 95% (0.96–1.94)).

Similarly, the total effect of compassion on work engagement was positive (β = 0.31, *p* < 0.01; C.I. 95% (0.54–1.59)), whereas it reported a not statistically significant direct effect on working environment fit (β = 0.10, *p* = 0.34, C.I. 95% (−0.19–0.60)). Interestingly, the decomposition of the total effect suggested that the association between compassion and work engagement was mainly accounted for the direct effect (β = 0.20, *p* < 0.05; C.I. 95% (574–1.47)) rather than for the indirect effect (β = 0.11, *p* = 0.33; C.I. 95% (−0.20–0.54)).

With regards to the association with working environment fit, data revealed a not statistically significant direct effects of subjective happiness (β = 0.05, *p* = 0.083; C.I. 95% (0.03–0.697)) and a statistically significant total effect of compassion (β = 0.20, *p* < 0.05; C.I. 95% (0.15–0.94)) on working environment fit. The analysis of indirect effects revealed the role of proactive strategies in mediating the association of subjective well-being and working environment fit. In fact, an indirect statistically significant effect was found in correspondence to subjective happiness (β = 0.43, *p* < 0.05; C.I. 95% (0.15–0.94)) meaning that in order to happiness to have an association with fit, teachers need to use their proactive strategies. A contribution to further understanding the role of proactive strategies was provided by the analysis related to the second hypothesis.

In general, the analysis of direct effects revealed that both subjective happiness (β = 0.57 *p* < 0.01; C.I. 95% (1.95–2.29)) and compassion (β = 0.15 *p* < 0.05; C.I. 95% (0.23–0.61)) were directly associated with proactive strategies, although with different magnitude. These results confirmed that the two correlated (*r* = 0.46) teacher’s well-being and compassion measures partially explain (*R*^2^ = 0.36) levels of proactive strategies (H2).

The last hypothesis was that proactive strategies adopted by teachers were associated with both work engagement and perceived teacher working environment fit with the professional community (H3). Analysis of direct effects suggested that proactive strategies were positively associated to work engagement (β = 0.55, *p* < 0.01; C.I. 95% (0.56–0.94)) and working environment fit (β = 0.77, *p* < 0.01; C.I. 95% (0.47–0.72)). Data supported that proactive strategies were more associated to recognition (β = 0.62, *p* < 0.01; C.I. 95% (0.45–0.73)) than to climate evaluation (β = 0.50, *p* < 0.01; C.I. 95% (0.42–0.72)). The model supported the idea that early childhood teachers’ self-regulation and coregulation (i.e., the ability to identify and use resources for coping with stressors as well as to seek and receive social support from colleagues) promote teacher working environment fit and work engagement.

All in all, the results showed that both early childhood teachers’ subjective happiness and compassion had reported, through the role of proactive strategies, statistically significant effects on work engagement and environment fit. From this point of view, proactive strategies reported a pattern of association that might be easily interpreted in line with full mediation analysis framework (Baron and Kenny, 1986) suggesting that the use of strategies was able to mediate the direct effects of both happiness and compassion on perceived teacher working environment fit.

## 4. Discussion

The aim of the present study was to advance the understanding of the interplay between subjective happiness, compassion, work engagement, experienced work environment fit, and proactive strategies (self-regulation and coregulated) in a group of kindergarten teachers. Although subjective happiness and compassion directly affected work engagement, their association with experienced work environment fit was found to be mediated by the use of proactive strategies. Statistical analysis of the proposed model revealed that self-regulation and coregulation (i.e., identifying and deploying resources for coping with stressors as well as seeking and receiving social support from colleagues) enhanced kindergarten teachers’ work environment fit and work engagement. Interestingly, self-perceived fit (which includes receiving professional recognition from colleagues and experiencing one’s community of teaching peers as constructive and empowering) was found to be more strongly associated with teachers’ coregulation than with their self-regulation. We now discuss the different roles played by the key variables under study, in relation to their direct and indirect effects on the teachers’ levels of work engagement and work environment fit.

### 4.1. The Direct and Indirect Effects of Subjective Happiness and Compassion on Work Engagement

Results suggest that when kindergarten teachers are disposed to experience happiness and compassion, they are more likely to actively construct an engaging work environment for themselves, by better managing their personal workload and the related emotions, as well as by developing mutually supportive relationships with colleagues.

Our data confirm earlier findings in the literature concerning, on the one hand, the relationship between positive emotions (triggered by subjective happiness and compassion at work) and proactive strategies and, on the other hand, the link between proactive strategies and personal involvement at work. For example, the results from our sample are consistent with previous evidence of the beneficial effects of positive emotions on individuals’ behavioral and cognitive repertoires [26], as well as the effects of personal and relational proactive strategies on work engagement [80].

Given that work engagement is the opposite pole of burnout, the current findings also bear out the findings of studies that identified subjective happiness and compassion as crucial personal resources for mitigating work-related stress and burnout [45,46,47]. Conversely, past researches has also documented a negative relationship between symptoms of depression and work engagement [81], thus indirectly confirming the key contribution of subjective happiness to work engagement. Indeed, happiness has been found to enhance both teaching and life experience, in general, and to increase work engagement by encouraging a more effective deployment of job resources [82]. However, as far as we are aware, subjective happiness has not previously been examined as a potential antecedent of work engagement among kindergarten teachers.

Our results also suggest that compassion influences work commitment. Again, this outcome is in line with a few previous studies that showed compassion to be correlated with work engagement [34,83,84]. In seeking to explain this association, Tremblay and Messervey [85] argued that compassion may generate work engagement by buffering against the impact of job demands on job strain. More generally, personal resources, such as subjective happiness and compassion, may shape individuals’ perceptions of their job resources which, in turn, are known to strongly predict work engagement [86,87].

### 4.2. The Direct and Indirect Effects of Subjective Happiness and Compassion on Experienced Work Environment Fit

Compassion and subjective happiness, contrary to our research hypothesis, had no significant direct effect on either of the two core dimensions of work environment fit, namely, perceived positive work climate and perceived recognition from peers and head teachers.

Our results suggest that subjective happiness and compassion alone are not enough to enhance kindergarten teachers’ work environment fit.

Analysis of the indirect effects within our statistical model showed that subjective happiness and compassion wielded significant indirect effects on the kindergarten teachers’ perceived work environment fit via the deployment of proactive strategies.

It appears that when early childhood teachers are happier and more compassionate, they are more likely to actively construct a good work environment fit, by managing their personal workload and related emotions more effectively, as well as by exchanging support with colleagues.

In other words, the psychosocial dynamics of the preschool setting should be taken into account when observing the relationship between kindergarten teachers’ subjective happiness and compassion on the one hand and their levels of organizational fit on the other.

This outcome is borne out by studies [88,89] that have documented the role of the specific work environment in determining person–organization fit as well as the salience of the organizational dimension in person–environment fit [90].

However, to more fully explain our findings, we need to take into account the nature and setting of kindergarten teachers’ work.

Specifically, kindergarten teachers are required to work as teams to organized and schedule educational activities. This means that they are called to continuously interact with colleagues, as well as with the other actors in their work environment, including the children’s parents, who rely on them for direct everyday contact with the preschool service [91]. Existing studies have shown that the highly relational nature of the early childhood education setting has key implications for teachers’ well-being and adjustment at school [91,92,93].

This might explain why we did not find compassion and subjective happiness to have a direct effect on organizational fit but rather to be mediated by teachers’ proclivity to adopt proactive strategies. As reported by Staw and Cohen-Charash [94], indeed, organizations may provide “strong situations” in which workers have higher chances to let their beliefs and dispositions arise and express. The role of organization may be meaningful for kindergarten teachers, as several studies have reported the collaboration within kindergarten staff (as well as with families and the broader social context) as being crucial to effectively pursue educational objectives [91,92,93,95,96]. Similarly, the effect of compassion and subjective happiness on work engagement was significantly higher when mediated by coping strategies.

These findings confirm the role of proactive strategies as protective factors, bearing out previous research which indicated that the capacity to use multiple coping strategies reduces emotional exhaustion within communities of teachers [97,98,99,100].

Overall, our results suggest that perceived work environment fit may be viewed as a social outcome that depends on the levels of happiness experienced by teachers at school; besides, it can be enhanced when members of teaching staff engage in self-regulated and coregulated strategies. In other words, proactive strategies that allow teachers to simultaneously regulate their own behavior and their work environment are an effective means of improving work environment fit.

## 5. Study Limitations and Future Perspectives

Some limitations of the present work should be mentioned. The data are cross-sectional, and therefore, it is not possible to draw inferences about cause-and-effect relationships. Thus, future researchers could use a longitudinal design to test the causal relations among variables, which might help us understand how relationships between them unfold over time. In addition, social desirability may have biased the results and our findings, and reliability and validity of our self-report measures may be impacted with regard to the translation of the instruments from English to Italian. Finally, it is not possible to generalize the findings to teachers located in cities or who are from different cultural backgrounds. Consequently, diverse samples should be used to test the generalizability of our findings in the future.

## 6. Conclusions

The current investigation contributes to shed new light on kindergarten teachers’ work engagement and experienced working environment fit by considering the contributions of subjective happiness, compassion, and proactive strategies to work engagement and perceived working environment fit in a single model.

The study’s findings indicate that subjective happiness and compassion at work can promote feelings which generate positive teachers’ attitudes and better work outcomes. It has to be noted that in order to make teachers feel more involved in their job and increase their perceived working environment fit, subjective happiness and compassion require to be transformed from personal disposition to proactive strategies with colleagues, i.e., supportive behaviors within the school community. This paper identifies the importance of proactive strategies upon teacher-experienced working environment fit. It is feasible to argue that through specific training actions, the increasing explicit kindergarten teacher awareness of their use of proactive strategies enables them to take responsibility for a better work environment fit. The employment of enabling strategies will differ according to personal, collective, and contextual needs.

Furthermore, these findings suggest some implications for kindergarten teacher education. Considering the emerged role of collaboration, training student teachers to work within teams (when taking decisions, solving problems, planning educational task), as well as to be aware and foster their interpersonal skills, could be useful to prepare them to face the school context. Studies addressing these dimensions have shown that when preservice teachers take part to preparation programs fostering these dimensions are better at dealing with interpersonal conflicts, empathetically relate to children, families, and colleagues, and better collaborate with other professionals [101,102].

The current data show that the early childhood teachers considered are more inclined to increase their working environment fit by drawing on positive, context-related relationships than by relying on their individual characteristics. Ultimately, the study’s findings suggest the importance of investing in the quality of the working environment. Positive interpersonal relationships experienced in the school context can promote early childhood teachers work engagement protecting them from the risk of work-related burnout [103,104,105,106,107,108,109].

It is well established that the development of the professional community is strongly dependent on the leadership strategies and, in the end, reflects the way the teaching profession is understood by the school community: leadership facilitating joint work is required for teachers to be positive about and effective in being more collaborative with their colleagues.

Based on these findings, we strongly advocate that educational policy makers and head teachers’ pay close attention to the areas of personal and collective resources and work-related well-being, with a view to effectively address the promotion of early childhood teachers’ work engagement and working environment fit.

## Figures and Tables

**Table 1:** Main descriptive statistics of variables.

Variables	M	SD	Skewness	S.E.	Reliability (α)
Subjective happiness	22.37	3.65	−0.562	0.143	0.751
Compassion	28.73	3.94	−0.769	0.143	0.872
Self-regulation	22.34	3.31	−0.564	0.143	0.691
Coregulation	17.97	2.18	−0.993	0.143	0.774
Work engagement	82.50	13.02	−0.460	0.143	0.934
Climate	14.93	3.88	−0.631	0.143	0.698
Recognition	16.83	3.14	−1.064	0.143	0.711

*Note.* M = Mean; SD = Standard deviation; S.E. = Standard error.

**Table 2:** Bivariate correlations between variables.

Variables	1	2	3	4	5	6	7	8
1. Age	–	0.26 **	0.09	0.00	0.02	−0.06	0.07	0.011 *
2. Seniority		–	0.18 **	0.10	0.09	−0.03	0.10	0.08
3. Work engagement			–	0.48 **	0.51 **	0.37 **	0.38 **	0.40 **
4. Self-regulation				–	0.67 **	0.37 **	0.26 **	0.46 **
5. Coregulation					–	0.47 **	0.27 **	0.42 **
6. Working environment fit						–	0.22 **	0.13 *
7. Compassion							–	0.33 **
8. Subjective happiness								–

*Note.* * *p* < 0.05; ** *p* < 0.01.
